# Gender Development in 46,XY DSD: Influences of Chromosomes, Hormones, and Interactions with Parents and Healthcare Professionals

**DOI:** 10.6064/2012/834967

**Published:** 2012-09-19

**Authors:** Amy B. Wisniewski

**Affiliations:** Department of Urology, The University of Oklahoma Health Sciences Center, Oklahoma City, OK 73104, USA

## Abstract

Variables that impact gender development in humans are difficult to evaluate. This difficulty exists because it is not usually possible to tease apart biological influences on gender from social variables. People with disorders of sex development, or DSD, provide important opportunities to study gender within individuals for whom biologic components of sex can be discordant with social components of gender. While most studies of gender development in people with 46,XY DSD have historically emphasized the importance of genes and hormones on gender identity and gender role, more recent evidence for a significant role for socialization exists and is considered here. For example, the influence of parents' perceptions of, and reactions to, DSD are considered. Additionally, the impact of treatments for DSD such as receiving gonadal surgeries or genitoplasty to reduce genital ambiguity on the psychological development of people with 46,XY DSD is presented. Finally, the role of multi-disciplinary care including access to peer support for advancing medical, surgical and psychosexual outcomes of children and adults with 46,XY DSD, regardless of sex of rearing, is discussed.

## 1. Introduction

For the past 2 decades, medical and surgical treatment approaches for managing 46,XY disorders of sex development (DSD) have received intense scrutiny: first from patient advocates followed by healthcare providers, researchers, and academics [1–7]. A combination of improved understanding of the impact of early hormone exposure on behavior, accrual of less-than-satisfactory gonadal and genital surgery outcomes data, and the formation of advocate groups demanding change in standards of medical and surgical care has resulted in disagreement among physicians about optimal treatment options for patients [8, 9]. For example, confusion exists about recommendations to parents of young children with DSD, as well as older affected individuals, concerning whether or not to proceed with medical therapies such as hormone replacement, surgical treatments such as gonadectomy and genitoplasty for ambiguous genitalia, and discussing one's DSD diagnosis and associated treatment with family and community members and/or joining support groups [10–13].

A critical evaluation of what is known about behavioral development, specifically psychosexual development, in people with 46,XY DSD born with female or ambiguous external genitalia is offered here. Consideration for hormonal, surgical and wider societal influences on behavior of affected individuals is included. A clearer understanding of psychosexual development in people with 46,XY DSD, coupled with an appreciation of the factors that influence development of behaviors important to this group, will make it is possible to resolve some of the disagreement and confusion experienced by patients, their families, and health care providers concerning optimal medical and surgical treatment choices. 

## 2. What Is 46,XY DSD?

DSD results when discordance between a person's genetic sex (XX or XY), gonadal sex (testes or ovaries), external genital sex (vulva or penis and scrotum), and/or internal sex ducts (Müllerian or Wolffian) exists at birth [9, 10]. While several subcategories of DSD are recognized based largely upon a person's sex chromosome complement, individuals with DSD including a 46,XY karyotype are the focus here and are referred to as having 46,XY DSD. However, people can also be affected by 46,XX DSD and sex chromosome DSD, although information about gender development in these groups is not considered here. People with 46,XY DSD exhibit a variety of phenotypes at birth ranging from typical female external genitalia, to ambiguous, and even underdeveloped male external genitalia including proximal or perineoscrotal hypospadias and/or bilateral cryptorchidism [9]. The emphasis of this review is on patterns of gender development and consideration of variables that influence these patterns in people with 46,XY DSD born with female or ambiguous external genitalia. 

To understand 46,XY DSD it is necessary to first appreciate processes underlying male sex determination and differentiation [10]. In humans, bipotential gonadal tissue differentiates into testes if a gene called *SRY* is present and functioning. Usually, *SRY* is encoded on the Y chromosome; however, this gene can be translocated to other chromosomes in some people. However, individuals who possess a Y chromosome including the *SRY* gene usually develop testes. In a typical scenario of fetal development, sex determination refers to an individual's genetic sex (i.e., 46,XY), while sex differentiation refers to testicular development from bipotential gonadal tissue. Once testicular differentiation takes place, the internal sex ducts and external genitalia will then develop in a male-typical fashion. Subsequent differentiation of the internal sex ducts and external genitalia are then driven by fetal hormones produced by the newly formed testes [9].

Müllerian inhibiting substance (MIS), also known as Müllerian inhibiting factor (MIF) or anti-Müllerian hormone (AMH), is a peptide hormone synthesized by the sertoli cells of the testes to inhibit development of female internal reproductive structures such as the fallopian tubes, uterus, cervix, and upper portion of the vagina. These structures will ultimately develop from the Müllerian ducts if not actively inhibited by this testicular hormone. All fetuses, regardless of the sex chromosome complement, possess Müllerian ducts initially so it is expected that the development of these structures will be inhibited in males as sex differentiation proceeds [10]. Simultaneously, leydig cells of the fetal testes produce the steroid hormone testosterone that is important for masculinization of male internal reproductive anatomy from the primitive Wolffian duct system. In the presence of testosterone, the Wolffian ducts (also present initially in all fetuses regardless of sex chromosome complement) develop into the epididymides, vas deferens, and seminal vesicles [9, 10]. Most 46,XY fetuses with testes produce MIF and testosterone and thus, inhibit female-typical structures from developing and also promote development of male-typical internal reproductive anatomy to correspond with their genetic sex and gonadal development. 

Finally, undifferentiated fetal external genital anatomy develops into a penis and scrotum when testosterone from the testes is metabolized into the potent androgen, dihydrotestosterone (DHT) [9, 10]. This is sometimes referred to as “delivery room sex” as parents are usually informed that they have a boy or a girl depending on the appearance of the external genitalia at birth. Local conversion of testosterone to DHT at the area of the developing external genitalia results in lengthening of a small phallic structure into a larger penis with a urethral meatus situated at the tip. DHT also causes the two distinct labial folds to fuse together and develop into a scrotum. Usually, the testes descend into the scrotum from the inguinal cavity just prior to, or soon after, birth. The ability to masculinize the external genital structures in males is a consequence of converting testosterone to DHT via the enzyme 5*α*-reductase as well as high affinity binding of DHT to androgen receptors (ARs) located in tissues comprising the phallus and labial folds that ultimately differentiate into the penis and scrotum. Thus, the external genitalia can serve as a bioassay for assessing early DHT production and action. Standards for penile length and width, as well as testicular volume exist for newborns ranging from 24 weeks to full-term gestational age. Such measures can be used to determine if sex determination and differentiation occurred in the expected manner for fetuses that possess a 46,XY chromosome complement and testes [9]. 

With the basics of male sex determination and differentiation presented, we can now begin to understand the six types of 46,XY DSD considered here. Generally speaking, 46,XY DSD results from 1 of 3 atypical physiologic scenarios. First, is the inability of target cells, such as those that exist in the tissues comprising the phallus and labial folds, to respond to androgens such as DHT. This inability of DHT (or any androgen) to bind to ARs is the mechanism underlying androgen insensitivity syndrome, or AIS. Such insensitivity is due to genetic mutations of the AR gene and can be complete (CAIS), resulting in a female genital phenotype including a small phallus/clitoris with a urethral meatus on the female-typical position of the perineum and 2 separated labial folds. Similar to the situation with typical male external genitalia, normative data for clitoral length, width, and anogenital distance for female genitalia are published [9]. AIS can also be expressed in a partial form (PAIS), resulting in an ambiguous genital phenotype including a phallus that is smaller than a typical penis but larger than a typical clitoris, a urethral opening located on the perineum or along the shaft of the phallus, and partially fused labial/scrotal folds. PAIS occurs when an AR gene mutation exists but androgens are able to exert limited physiologic effects on target tissues. Whether or not a person is affected by CAIS or PAIS has consequences for both their physical and behavioral development, as well as the medical and surgical treatments offered to them [14]. For some people with clinical signs of PAIS, but no identifiable AR gene mutation, fetal exposure to endocrine disrupting compounds has been implicated [15]. Although clinically similar to PAIS, these individuals are not androgen insensitive *per se* from a mechanistic standpoint.

A second cause of 46,XY DSD is the inability of the testes, as a result of one of several possible enzyme deficiencies, to produce hormones necessary for typical development of male external genital structures, Wolffian ducts, and/or suppression of the Müllerian ducts [16–18]. Examples of this group of 46,XY DSD include 5*α*-reductase type 2 (5*α*-RD2) and 17*β*-hydroxysteroid dehydrogenase-3 (17*β*HSD-3) deficiencies. For genetic males with these conditions, the external genital phenotype at birth can be female typical or ambiguous. The gonads differentiate into testes in both conditions, Wolffian duct development can be male typical due to testosterone production by the leydig cells of the testes, and the Müllerian ducts are inhibited due to MIF production by the sertoli cells of the testes [17]. Individuals affected by 46,XY DSD including female external genitalia are more likely to possess identifiable genetic lesions than their counterparts born with ambiguous genitalia when the underlying cause is a biosynthetic defect of testicular hormone production during fetal development [18]. People with either of these conditions can experience the most variable patterns of psychosexual development observed in 46,XY DSD, as discussed in greater detail in the following sections. 

Finally, 46,XY DSD can result from the inability of the bipotential gonads to differentiate, either completely or partially, into testes during early fetal development. This situation is referred to as gonadal dysgenesis and ultimately results in decreased testicular hormone production. In these cases, the problem does not result from an enzyme deficiency, but rather is due to failure of the leydig and/or sertoli cells to form properly. Mutations or copy number variations of several sex-determining genes, including but not limited to *SRY*, *NR5A1*/*SF1*, *DAX1*, *WT1*, *SOX9*, *MAP3K1*, and *GATA4*, result in gonadal streaks or dysgenetic testes in people with a 46,XY karyotype [19–27]. As most people with 46,XY DSD do not possess any of these known mutations, more genes needed for development of a bipotential gonad into subsequent testicular differentiation are certain to be identified with time.

One of the many challenges to understanding the natural history of psychosexual development in people with 46,XY DSD, regardless of the appearance of their external genital phenotype at birth, is that these conditions are heterogenous in nature and only a fraction of cases can be attributed to known genetic causes at this time. Thus, if people with 46,XY DSD due to certain gene mutations or enzyme deficiencies are predisposed to unique psychosexual trajectories, then this may be underappreciated as long as causes for DSD remain unidentified. New technologies for understanding the multiple etiologies of 46,XY DSD, such as comparative genomic hybridization, custom array sequencing and next generation sequencing may result in greater understanding of the genetic causes of DSD for patients who possess a Y chromosome [28, 29]. However, these improvements have not yet been realized clinically. Thus, when considering behavioral development in people with different types of 46,XY DSD, it is often necessary to group individuals according to clinical diagnoses based on anatomy and hormone levels but unconfirmed by molecular evaluation. As discussed below, patients are often grouped together for study if they are born with female versus ambiguous external genitalia despite the fact that their DSD may result from any number of causes ranging from target tissue insensitivity to androgens, to androgen biosynthetic defects due to enzyme deficiencies, to gonadal dysgenesis. 

## 3. Psychosexual Development

Psychosexual development includes, but is not limited to, gender identity (GI) and gender role (GR). GI refers to self-identification as male, female, or intersex [30]. In other words, a person with a female GI views themselves as a woman. GR refers to overt behaviors that an individual's society or community considers masculine or feminine [31]. For example, a person who enjoys hunting or participating in contact sports such as American football would be considered by many, but certainly not all, groups of people. GI is the most important component of psychosexual development to consider when determining a sex of rearing for newborns with 46,XY DSD. In people not affected by 46,XY DSD, identifying with others of the same sex and exhibiting gender-typical behavior is positively associated with adjustment and self-esteem [32, 33]. In people with DSD, having a GI that is congruent with sex of rearing is also associated with positive social and emotional development. In contrast, discordance between GR and sex of rearing appears to be less important [9, 10]. Understanding long-term psychosexual development in people with 46,XY DSD is paramount for optimizing quality of life and psychological outcomes for patients diagnosed in infancy for whom genital ambiguity exists [3, 4, 8–10]. 

Measuring psychosexual development in people with DSD is difficult, in part due to the fact that gender assessment tools are few in number and often come with methodological limitations [34]. For example, patients may provide inaccurate answers on questionnaires to appease their parents' or doctors' expectations of their behavior, rather than reflecting their actual experiences with GI and GR. Additionally, parents may report biased data about their affected child's gender to meet their own expectations about their son's or daughter's behavior as well as those expectations of researchers and physicians. Alternatively, people with 46,XY DSD may hide their gender-atypical behavior from their family and peers. Although many measures of psychosexual development in people are limited by these constraints, most data indicate that social influences such as parents' reactions to DSD as well as which sex they choose to rear their child as impacts the formation of GI; [35] whereas biological influences such as early androgen exposure along with social influences or learning exert a strong influence on GR development [36, 37]. Thus, to fully understand human psychosexual development in the context of 46,XY DSD both nurture and nature need to be taken into account [38–42]. 

Psychosexual development evolves over time, and a person's age is another important factor to consider when studying gender [43]. This means that any optimal assessment tool for measuring GI and GR will need to be validated for children, adolescents, and adults across the developmental spectrum. Current data reveal that for some types of 46,XY DSD, psychosexual development is condition specific and perhaps more strongly influenced by genes and hormones; [44, 45] however, for other types of 46,XY DSD factors such as sex of rearing are paramount [43, 46]. Improvement in our ability to determine which patients' gender development is more heavily influenced by genes and hormones (nature) versus learning and socialization (nurture) is needed. With better assessments of psychosexual development in people with 46,XY DSD, it may be possible to reduce the number of gender assignments that prove to be incongruent with ultimate GI development in affected people [9]. 

Because of the previously mentioned limitations to using questionnaires and parent observations for determining GI and GR in people with 46,XY DSD across the lifespan, more objective measures of gender are desired. One possibility is to identify an anatomical or physiological measure of the nervous system that reliably predicts gender development. Ideally, such a measure would be present at birth and be used as part of the clinical algorithm for assigning sex, when appropriate, for newborns with 46,XY DSD. Two such neurophysiologic measures that may meet these requirements are otoacoustic emissions (OAEs) and auditory evoked potentials (AEPs). Specifically, OAEs are sounds generated by the cochlea [47] and are both more numerous and stronger in females compared to males [48–50]. AEPs are sound-induced electrical responses produced by the auditory cortex in response to auditory stimuli. Both OEAs and AEPs are sexually dimorphic in newborns, [51] suggesting that they are more heavily influenced by biological variables (genes and/or prenatal androgen exposure) rather than socialization or learning associated with sex of rearing. 

A good deal of evidence exists in nonhuman animal species for early androgen exposure to influence sexually dimorphic patterns of OAEs and AEPs. For example, female-spotted hyenas (*Crocuta crocuta*) are normally exposed to high androgen levels during their prenatal development. OAEs among females of this species are masculinized (i.e., weakened) as predicted if early androgen exposure does in fact influence sexually dimorphic patterns of these components of the nervous system. Additionally, female hyenas treated with antiandrogens during their gestation exhibit female-typical (i.e., stronger) patterns of OAEs [52]. Studies of people with 46,XY DSD stratified according to their sex of rearing and degree of prenatal androgen exposure, as determined by the presence of female versus ambiguous genitalia at birth, would provide an excellent opportunity to determine if OEAs and AEPs are both sensitive to prenatal androgen exposure and are also predictive of GI development in people with 46,XY DSD. 

In addition to the sex differences in patterns of OAEs in human infants noted above, relationships between gender, OAEs and AEPs exist in adult non-DSD populations. For example, homosexual and bisexual women exhibit OAE and AEP patterns that are masculinized or shifted in the male-typical direction of being weakened [50, 53]. These data indicate that both the cochlea and auditory cortex are masculinized in women not affected by DSD who exhibit male-typical patterns of gender development (i.e., sexual attraction toward females). Thus, if these measures predict GI in a similar manner to how they predict sexual orientation, then it may be possible to employ these auditory measures from very early in development to predict optimal sex of rearing in people with 46,XY DSD reared male or female. If this turns out to be the case, then these aspects of the auditory system may one day be considered as part of an algorithm for planning medical and surgical management for newborns with 46,XY DSD along with the currently relied upon genetic, anatomic, and hormonal evaluation. 

## 4. GI in Major Subcategories of 46,XY DSD

Here we consider how the etiology of 46,XY DSD, including timing and type of hormone exposure as well as social factors such a family and peer influences impact the aspect of psychosexual development referred to as GI for affected individuals. As previously described, AIS refers to a genetic condition in which target tissues are unresponsive to all androgens, and this insensitivity can be complete (CAIS) or partial (PAIS). AIS results from mutations of the AR gene found on chromosome Xq11-12, thus only individuals with a 46,XY chromosomal complement are affected. People with a 46,XX chromosomal complement can be carriers for AIS, and this DSD is often transmitted through the maternal line. Genetic females who are carriers for AIS possess an AR gene mutation on only one of their two X chromosomes, so they are able to respond to androgens as a result of inactivation of the X chromosome that encodes the mutation. People affected by CAIS are born with testes but also typical-appearing female external genitalia, and no Müllerian structures, whereas those with PAIS have testes, ambiguous external genitalia, and no Müllerian structures [54]. The most recent update of AR gene mutations reveals that more than 1,000 have been identified in humans to date, [55] but also that a significant number of people with CAIS or PAIS do not possess a gene defect that is detectable with current technology [56]. 

People with CAIS are almost universally reared as girls as a combined result of their female external genital appearance at birth, diagnosis in later childhood or adolescence when testes are detected or primary amenorrhea is noted, and the overwhelming number of reports in the scientific literature describing a female GI for the vast majority of affected individuals [57–62]. While a small number of reports of people with CAIS who develop an ambiguous or male gender identity exist, [63, 64] these are highly unusual manifestations of psychosexual development in people with this particular type of 46,XY DSD [57]. Many variables, both biological and social, likely contribute to the overwhelming preponderance of female GI development in the context of CAIS. For example, because people with this condition are completely insensitive to all androgens, this means that their brains cannot be directly masculinized by testosterone or DHT exposure at any point in their development despite the high levels of both of these androgens circulating in the bloodstream. Additionally, because affected individuals look no different than girls who do not have CAIS, along with their typically later diagnosis in childhood or adolescence, family members and peers treat affected girls no differently than unaffected girls for most of their early development. Thus, socialization proceeds in a female-typical manner. 

In contrast to the near ubiquitous female rearing and GI observed in girls and women with CAIS, more people with PAIS are raised male and correspondingly develop a male GI [65, 66]. Additionally, people with PAIS are more likely than those with CAIS to identify with a gender that is incongruent with their sex of rearing—regardless of whether their sex of rearing was male or female. Once again, both hormonal and social variables likely impact the fact that GI development is less predictable for people with PAIS compared to those affected by CAIS. People with PAIS are able to physiologically respond, albeit to a limited degree, to testosterone and DHT exposure during fetal development so that their external genitalia are neither female nor male at birth, but are instead ambiguous. This means that the brains of people with PAIS are also exposed to, and respond to, androgens early in life—although to a greater extent than is the case for unaffected females and to a lesser extent than is the case for unaffected males. Furthermore, because of their phenotypic ambiguity at birth, people with PAIS are usually diagnosed very early in life and may not be treated as completely male or completely female by family and peers from the very start of their development. Such treatment could result in an ambiguous socialization of their GI or even a lack of commitment to treating affected people as one sex or the other by family and community members. Although GI development in people with PAIS is more variable than in people with CAIS, the best predictor of GI in people with PAIS is also sex of rearing [65]. Thus, the amount of fetal androgen exposure experienced by people with this type of DSD, although important to physical and some behavioral development, is insufficient to completely override the impact of learning and social experiences on GI for people with this particular type of 46,XY DSD [63, 66, 67]. 

An exception to the observation that sex of rearing and socialization by parents and peers are the primary influences on GI in people with 46,XY DSD are the frequent reports of male GI development despite female rearing documented in adolescents with a 46,XY chromosomal complement and 5*α*-RD2 deficiency [68–71]. As previously mentioned, 5*α*-reductase is the enzyme required to synthesize DHT from the precursor hormone testosterone. Fetuses that do not produce 5*α*-reductase cannot convert testosterone to DHT during the critical period when differentiation of the external genitalia occurs. Consequently, their external genitalia look female typical or only mildly masculinized at birth. However, people with 5*α*-RD2 deficiency do produce and respond to testosterone no differently than unaffected males, and they will experience virilization from puberty onward if their testes remain *in situ*. 

17*β*-hydroxysteroid dehydrogenase-3 is the enzyme needed to produce testosterone from the precursor hormone androstenedione. Genetic males with 17*β*HSD-3 deficiency cannot convert androstenedione to testosterone. Like 5*α*-RD2 deficiency, people with 17*β*HSD-3 deficiency are also born with female or mildly masculinized external genitalia despite having a 46,XY chromosomal complement and possessing testes. Also similar to 5*α*-RD2 deficiency, genetic males with 17*β*HSD-3 deficiency also exhibit an increased incidence of developing a male GI by adolescence despite their initial female rearing [41, 71]. People with either of these conditions in which the biosynthesis of androgens is inhibited due to an enzyme deficiency may have incomplete or full Wolffian duct development and no Müllerian structures. At puberty, virilization occurs from androgens produced by the testes if they remain *in situ *or from exogenous testosterone therapy. Why people with 46,XY DSD due to testosterone biosynthetic defects are more likely to develop a male GI than those with CAIS or PAIS is not fully understood at this time, although it seems probable that androgen exposure at puberty exerts an impact. 

Fewer reports of gender development exist for individuals with 46,XY DSD due to gonadal dysgenesis compared with either AIS or enzyme deficiencies associated with androgen biosynthesis. In the complete form of gonadal dysgenesis, also known as Swyer syndrome, female external genitalia coupled with streak gonads, fully formed Müllerian ducts, and underdeveloped Wolffian duct structures exist in a person with a 46,XY chromosomal complement. The lack of production of MIF, testosterone, and DHT from the streak gonads of people with Swyer syndrome explains the reproductive anatomy associated with this condition. For some, but not all, people with Swyer syndrome, the underlying etiology is a mutation or deletion of the *SRY* gene necessary for differentiation of a bipotential gonad into a testis [72–75]. As a result of possessing Müllerian duct structures, 46,XY individuals with Swyer syndrome have successfully carried pregnancies and given birth to healthy children with the help of assisted reproductive technology [76, 77]. Similar to CAIS, women with Swyer syndrome are universally reared female and develop a female GI; [78] however, the latter group has not been studied as thoroughly from a psychological perspective. Influences on female GI in people with Swyer syndrome include having no testicular hormone production or action during fetal development, a female genital phenotype at birth coupled with a diagnosis in later childhood or adolescence when puberty fails to occur spontaneously. 

Partial gonadal dysgenesis in an individual with a 46,XY chromosomal complement leads to bilateral dysgenetic testes during fetal development that are unable to produce male-typical amounts of androgens and Müllerian inhibiting substance (MIS) from the leydig and sertoli cells, respectively. The result of partial gonadal dysgenesis is ambiguous external genitalia coupled with partial development of both Müllerian and Wolffian duct structures. Similar to Swyer syndrome, partial gonadal dysgenesis can result from mutations of the *SRY* gene [79]. Additionally, dosage imbalances and mutations of *DAX1* [80], *DMRT1* [81], and *MAP3K1* [20] have been documented. The genital phenotype of affected newborns can resemble that of newborn with ambiguous genitalia due to PAIS, 5*α*-RD2, or 17*β*HSD-3 deficiencies; however, serum MIS levels effectively distinguish between these different causes of 46,XY DSD if this peptide hormone is measured at appropriate times [82]. Like PAIS, the best predictor of GI in people with partial gonadal dysgenesis is sex of rearing [65, 83]. Also like PAIS, GI is discordant with sex of rearing for a significant minority of affected individuals, whether raised male or female [65, 83]. As is the case with PAIS, both hormonal and social variables surely impact GI development in people with partial gonadal dysgenesis. Affected individuals are able to respond to a limited degree to testosterone and DHT exposure during fetal development so that their external genitalia are neither female nor male at birth. This means that their brains are also exposed to, and respond to, androgens early in life albeit to a greater extent than is the case for unaffected females and to a lesser extent than is the case for unaffected males. Ambiguous external genitalia of people with partial gonadal dysgenesis is evident at birth so that an ambiguous socialization of GI or a lack of commitment to treating affected people as one sex or the other by family and peers may occur.

When considered together, the data reveal that GI development usually corresponds with sex of rearing for people with 46,XY DSD including female (CAIS and Swyer syndrome) or ambiguous (PAIS and PGD) external genitalia. Thus, despite different exposure histories to testicular hormones such as DHT, testosterone, and MIS, people with these forms of DSD typically develop a female GI when reared as girls and a male GI when reared as boys. Thus, learning and nurture appear to be paramount for GI development in people with these conditions. In contrast, people with 46,XY DSD due to 5*α*-RD2 or 17*β*HSD-3 deficiencies and reared female often develop a male GI, and it is not understood at this time why people with 46,XY DSD due to enzymatic defects experience diverse trajectories in their psychosexual development [16]. Perhaps parents, siblings, and peers treat people with 5*α*-RD2 or 17*β*HSD-3 deficiencies differently than those with AIS or gonadal dysgenesis? As many people with these enzymatic defects have normal-appearing female external genitalia at birth, this explanation seems unlikely. Perhaps there are other societal or hormonal factors that have not yet been appreciated that uniquely influence GI development in these 2 subgroups of 46,XY DSD? Physiologic commonalities between 5*α*-RD2 and 17*β*HSD-3 deficiencies that may contribute to a predisposition to male psychosexual development include male internal sex duct development that does not differ from unaffected males as well as an alternate biosynthetic pathway for producing testosterone at puberty [84]. While the mechanisms underlying GI development are unclear for 5*α*-RD2 or 17*β*HSD-3 deficiencies, it is known that male rearing is preferable for babies categorized into this subgroup of 46,XY DSD due to their increased likelihood of developing a male GI. Furthermore, affected individuals reared male who retain their testes have fertility potential as males, but not as females, and do not require testosterone therapy to induce puberty and maintain secondary sexual characteristics in adulthood [68]. 

## 5. GR in Major Subcategories of 46,XY DSD

GR includes overt behaviors such as toy preference, playmate choice, and occupational interests that differ between the sexes. Unlike GI, GR appears to be more heavily influenced by early hormone exposure than learning or socialization [85, 86]. Most studies of GR in people with DSD have focused on girls and women with 46,XX DSD due to the condition congenital adrenal hyperplasia (CAH) [87–90]. Relatively few investigations of GR have been conducted in people affected by 46,XY DSD. In one such study of children ages 2–12 years, parents reported that their daughters with CAIS exhibits GR behaviors no different from unaffected girls [37]. These results are in agreement with self-reported feminine behavior by girls and women with CAIS across their lifespan [57, 58]. In contrast to CAIS, girls with PAIS or other types of 46,XY DSD that include some early androgen exposure exhibit more masculine behavior and interests. However, girls who experienced early androgen exposure were also reported to be more feminine that their counterparts reared male [37]. Thus, both early hormonal exposure and socialization contribute to the development and expression of masculine and feminine patterns of GR in people with 46,XY DSD. 

Our group retrospectively studied GR across developmental stages in people with 46,XY DSD due to PAIS or partial gonadal dysgenesis [43]. People reared female reported increasing femininity and decreasing masculinity as they progressed from childhood to adolescence and adulthood. Those reared male reported increasing masculinity across development [43]. This pattern of results may reflect an additive impact of socialization by parents and peers on GR over time. Consistent with this theory are observations of boys and girls unaffected by DSD who also increasingly prefer same sex playmates [91] and gender typical toys as they age [92]. Alternatively, pubertal hormonal exposure during late childhood and early adolescence could also explain increasing femininity and masculinity in people with 46,XY DSD reared female or male, as well as in unaffected girls and boys. As affected children reared female experience a feminizing puberty while those reared male have a masculinizing puberty, it is impossible to distinguish between the independent influences of hormones and learning on GR development over time for people with or without 46,XY DSD from currently available data. 

## 6. Parental Influences on GI and GR in 46,XY DSD

Parent and family functioning predict psychological adjustment in children with all types of chronic medical conditions. For example, family cohesion and maternal psychological adjustment are both associated with fewer behavioral problems in children receiving ongoing medical care [93]. Despite the increasing evidence of an important role for parents and families for psychological development in children with chronic health conditions, few studies have focused on parental influences on gender development in people affected by 46,XY DSD. What is known about parents of children with DSD is that they often experience surprise and self-blame upon learning of their child's condition [94]. Parents of daughters with CAIS experience grief, anger, and shame upon learning of their child's condition, and mothers in particular feel guilt—undoubtedly due to the fact that CAIS is an X-linked DSD [95]. In children with chronic medical conditions other than DSD, paternal involvement can be secondary to the role that mothers play in children's health outcomes [96]. Although this is an important area of research that deserves further study, we do not have sufficient data at this time to understand if, or how, mothers and fathers differ in their influence on GI and GR development in children affected by DSD.

Parents can impact their affected child's psychosexual development in at least 2 important ways: (1) by the choice they make concerning the sex of rearing for their child along with associated medical and surgical treatment decisions, and (2) by what types of gendered behaviors they encourage or discourage for their sons and daughters. For example, some parents of children with DSD reinforce hobbies that correspond to their child's sex of rearing; [97] however, for girls with 46,XX DSD parents do report encouraging sex-atypical play [98]. Presumably, this is in response to their daughters' tomboyish interests that have been extensively documented for this group since the 1950s [9, 31, 84–90]. Far more data are available about parents' influences on gendered play behavior in children with 46,XX DSD compared to children with 46,XY DSD. Therefore, it is not known if the latter group of parents encourages sex-atypical play in their children, whether raised female or male. 

Parents often report a desire to medically and surgically reduce sexual ambiguity inherent to their child's DSD [99]. Parents also wish to maintain privacy and avoid negative social experiences for their child such as bullying that they anticipate will result when others learn about their child's sexual ambiguity [35, 99, 100]. Our data, collected from parents across the United States whose children attend one of several hospitals for DSD care reveals that mothers perceive their children with 46,XY DSD and raised male as particularly vulnerable [101]. This may be due to the fact that cosmetic outcomes of genitoplasty for young boys can be worse than for young girls, and boys may also undergo more genital surgeries than girls early in life [65]. Mothers also report greater parenting stress if their child with a DSD has not received genitoplasty to reduce their child's ambiguity [102]. Interestingly, although far fewer fathers participated in this research, those who did so reported more stress if their child received genital surgery early, specificially within the first year of life. 

In other words, parents view their children with DSD as vulnerable as a result of their sexual ambiguity, and they experience parenting-related stress associated with this vulnerability. These parents often desire to help their children become more sex typical in both their behavior and physical appearance in an attempt to protect them from maltreatment by others [100–102]. Additionally, mothers and fathers report different feelings associated with surgical treatments for ambiguous genitalia often received by children with DSD. Thus, we should not assume that all parents experience similar reactions to having a child with DSD, nor should we assume that all parents have similar opinions about the best course of action when deciding on a child's medical and surgical plan. 

A number of parents of children with DSD report clinically significant stress, and this stress can be related not only to medical and surgical management decisions as described above, but also to poor communication with healthcare professionals and other family members [99–103]. For example, many parents experience frustration while attempting to obtain information about DSD from their child's healthcare team [35]. A child's developmental stage can also impact how parents communicate with that child about their DSD and related care [103]. For example, parents of older children with 46,XY DSD experience significant stress when their communication with their children is problematic. Problematic parent-child communication may result from parents' unease with discussing sexuality and gender with their son or daughter as well as their discomfort with explaining their child's medical history to them. In contrast, parents of infants and toddlers with 46,XY DSD report more overprotective parenting of their children, perhaps in response to salient events that occur at these earlier times such as gender assignment and whether or not to proceed with genitoplasty. Thus, just as gender evolves over time for people with 46,XY DSD, parents' reactions to DSD also change in accordance with their child's growth and developmental stages. How, or if, parents' reactions to 46,XY DSD impact GI or GR development remains to be elucidated. However, it is clear that for people with 46,XY DSD due to causes other than 5*α*-RD2 or 17*β*HSD-3 deficiencies, sex of rearing by parents is a key factor for establishing GI and, to a lesser extent, GR development. 

## 7. Surgical Influences on Gender in 46,XY DSD

As already mentioned, some people with 46,XY DSD receive genitoplasty and gonadal surgeries, often at very young ages, as part of their medical assessment and surgical treatment. Such surgeries are performed for multiple reasons including to align a child's phenotype more closely with their sex of rearing, determine future fertility potential, and remove gonads that are malignant or at high risk for malignancy [10, 104]. Recent data have been provided to allow for a more accurate determination of malignancy risk in differentiated testes associated with 46,XY DSD, as well as for gonadoblastoma precursors in undifferentiated gonadal tissue for people with DSD who possesses a Y chromosome [105]. The result of this information has been greater acceptance of leaving the undifferentiated gonads or testes *in situ* with appropriate monitoring over time for certain individuals [104]. 

For girls with CAIS, the risk for testicular malignancy is low prior to adulthood, [105] and a spontaneous puberty will occur including optimal breast and bone development when the testes remain *in situ* to produce levels of androgens that are high enough to be converted to adequate estrogen levels for feminization. Because their target tissues are unresponsive to androgens, development of male secondary sex characteristics should not occur for these girls who retain their testes. Girls who experience this type of spontaneous, feminizing pubertal maturation report self-esteem that is higher than their counterparts who require pubertal induction with exogenous estrogens because their testes were surgically removed as young children [106, 107]. Whether these girls also have different experiences with their GI and GR development is not known. As reported cases of testicular malignancy do exist for younger girls with CAIS, although rare, it is important to regularly monitor the gonads of these patients with ultrasounds if they are undescended or with a manual exam if they are descended prior to their removal [108]. For people with 5*α*-RD2 or 17*β*HSD-3 deficiencies who are reared male, leaving the testes *in situ* impacts psychosexual development by reinforcing masculinity with both a virilizing puberty and maintaining male fertility potential [44, 109]. Individuals with these enzymatic deficiencies who possess testes should have regular testicular exams similar to unaffected males. For people with other forms of 46,XY DSD such as PAIS, partial gonadal dysgenesis, and Swyer syndrome, the gonads may need to be removed as individuals in these subgroups are considered to be at higher risk for gonadal malignancy [105]. 

Feminizing or masculinizing genitoplasty to construct genitals that appear and function in a more female- or male-typical manner also occurs as part of the medical management of 46,XY DSD [10, 104]. Feminizing surgeries for ambiguous genitalia include clitoroplasty and vaginoplasty. Although total excisions of the clitoris have been performed historically, this type of genitoplasty is no longer recommended. Rather, clitoroplasty that preserves the glans and neurovascular bundle of the phallus is the currently accepted approach for making a phallus resemble a clitoris as it is believed that this approach results in better genital sensation and orgasmic potential [110]. However, very few long-term studies comparing sexual function in women with DSD who received the more recent glans and nerve-sparing surgeries to their counterparts who received total excisions or to control women who received no clitoral surgery have been performed. As a result of this deficiency in data, disagreement exists as to whether of not even modern approaches to clitoroplasty are appropriate [9]. Regarding vaginoplasty, the type of procedure employed depends on the point of entry of the vagina into the urogenital sinus. Modern approaches to vaginoplasty include skin flap, sigmoid bowel, and pull-through procedures [10]. Similar to clitoroplasty, it is difficult to compare sexual function outcomes from people who received different types of vaginoplasty procedures as most studies are confounded by variation in patients' anatomy prior to their surgery as well as surgeons' experience and skill levels [110]. While we do not fully appreciate how receiving clitoroplasty and/or vaginoplasty impact GI and GR development, limited data indicate that patients with 46,XY DSD reared female who ultimately develop a male gender do not experience different cosmetic or functional outcomes from their genitoplasty than those who develop a female GI and GR [65, 111]. It is increasingly becoming apparent that calls for a reassessment of the appropriateness of proceeding with feminizing genitoplasty in young girls with 46,XY DSD is primarily a question of improving quality of life and sexual function for these people, and less of a concern that GI development will be discordant with their female sex of rearing [2–9, 112]. As more parents of girls with 46,XY DSD decide to postpone genitoplasty for their daughters until they can provide assent or informed consent for these procedures themselves, a clearer understanding of the impact of having or not having these surgeries on gender development may be possible. What is currently known is that some women who received early genitoplasty regret this experience, and they believe that such procedures should only be performed in older adults, if at all [112].

Masculinizing surgeries for ambiguous genitalia include release of ventral chordee, hypospadias repair, and placement of prosthetic testes in the scrotum at puberty if gonadectomy is indicated. Like vaginoplasty, the procedures used for hypospadias repair depend upon the child's anatomy including the size of the phallus, severity of the hypospadias, and availability of tissue for advancing the urethral meatus to the tip of the phallus [104]. Historically, it was believed that masculinizing genitoplasty required more procedures than feminizing genitoplasty and also resulted in a poorer cosmetic outcome [65, 112]. More recent data reveal that the number of surgical procedures used to construct male-appearing genitalia has decreased over time along with the number of surgical complications [104, 113]. While men who received such surgeries decades ago are generally dissatisfied with the appearance of their penis, [112] evaluation of cosmetic satisfaction among younger men who have received more modern surgical approaches remains to be conducted. Similar to girls and women with ambiguous genitalia, we do not fully appreciate how receiving masculinizing genitoplasty impacts GI and GR development in males born with 46,XY DSD. Also similar to their female counterparts, patients with 46,XY DSD reared male who ultimately develop a female gender do not experience different cosmetic or functional outcomes from their genitoplasty than those who develop a male GI and GR [65, 111]. 

 DSD patient advocates are increasingly emphasizing their decreased quality of life and poor sexual function that they attribute to their early genitoplasty outcomes; [2–12] thus, these topics deserve greater consideration by healthcare providers and researchers. Cosmetic and functional outcomes of these surgical procedures vary greatly between reports—even within specific diagnostic categories of 46,XY DSD. For example, recent investigations of outcomes of gonadectomy and vaginoplasty in girls and women affected by CAIS range from satisfaction with surgery, [114] to preference for early surgery, [115] to a lack of sexual desire/arousal and dyspareunia attributed to these procedures [116]. Thus, more study is needed to understand why such disparate results are observed for outcome studies of identical genitoplasty procedures among groups of people with the same type of 46,XY DSD. Perhaps individual differences in presurgical anatomy explain this variance. Alternatively, differences in experience and skill level between surgeons who perform these procedures may explain the inconsistent outcomes that exist in the literature. Finally, some parents may not receive adequate presurgical counseling and this could contribute to child's ultimate dissatisfaction with their genitoplasty outcomes. 

While outcome studies of feminizing genitoplasty outnumber those of masculinizing procedures, more surgical studies of people with 46,XY DSD reared male are being conducted. Although older male patients who received a high number of genitoplasty procedures as young boys report disappointing results, [116, 117] this scenario may be changing for younger boys and men who have received fewer surgeries with more modern approaches for changing the appearance of their ambiguous genitalia [104]. Despite older men's negative experiences with genitoplasty, they do not report more difficulties with either their psychosexual development [57] or their overall psychological adjustment [53] than women with 46,XY DSD who received on average fewer genitoplasty procedures associated with better cosmetic (although not necessarily functional) outcomes. Thus, possessing atypical-appearing genitalia may not pose as great of a risk to gender development as previously thought [9]. Additionally, undergoing more genitoplasty procedures does not necessarily lead to problems with psychosexual development or psychological adjustment; however, many patients report that their quality of life and sexual function are harmed [112]. 

## 8. Recent Changes to Improve Delivery of Care for 46,XY DSD

As already mentioned, medical and surgical treatment approaches for managing 46,XY DSD have recently received intense scrutiny, and as a result certain recommendations have been made for improving delivery of care to affected children and adults [1–12]. Changes in medical terminology to reduce stigmatization associated with DSD have been proposed [9] and largely accepted [118]. For example, the terms *male pseudohermaphrodite*, *undervirilization of an XY male*, and *undermasculinization of an XY male* have been replaced by *46,XY DSD*. 

Additionally, it was proposed that medical and surgical management of 46,XY DSD should be performed at medical centers with an experienced multidisciplinary team [9]. Although the team approach has been largely embraced by healthcare providers in Europe, [118] only a handful of centers in the United States have described protocols for delivering this type of care to their patients [104, 119]. While some evidence exists that multidisciplinary care results in better medical management and also an increased likelihood for interacting with others with similar medical histories for patients with 46,XX DSD, [120] this has yet to be demonstrated for 46,XY DSD. Multidisciplinary teams for delivering care is increasingly being demanded by people affected by 46,XY DSD, and advice for forming and maintaining such clinics is available [121, 122]. Professionals who contribute to such clinics adhere to the idea that the manner in which patients are supported and educated about their condition and medical/surgical history, including provision of peer support, optimizes quality of life for people with DSD [122]. Whether or not multidisciplinary care and interactions with peer support groups influences GI and GR development in significant ways is not known. It does appear that centers that offer multidisciplinary care for DSD perform fewer genital surgeries; [118] however, as described earlier genital surgeries do not appear to influence GI and GR development. We do know that parents of affected children and DSD patient advocates report greater gender-related concerns than the specialized physicians who provide them with medical and surgical care, indicating the importance of having input from patients and their family members on how to best deliver DSD care within a multidisciplinary team [123]. 

Finally, a reappraisal of the appropriateness of performing genital surgery on young children with ambiguous genitalia due to DSD is currently underway [2–8]. Controversy concerning such surgeries is contributed to several factors including the inability of young patients to provide informed assent or consent for these procedures, reports of poor long-term cosmetic and functional outcomes associated with genitoplasty as described earlier, and a frequent need for additional genital surgeries when the patient is ready to become sexually active in adolescence or adulthood [124]. Despite these concerns, many DSD specialists and affected children's parents have been reluctant to abandon early genitoplasty for treating ambiguous genitalia as it is not known what psychological and social risks a child with ambiguous genitalia faces [9, 125]. Among patients with 46,XY DSD including ambiguous external genitalia at birth and reared female, as well as among parents of such children, support for early genital surgery has been documented [105, 112]. Additionally, satisfaction of men with 46,XY DSD who received masculinizing genitoplasty procedures has also been documented, despite their voicing significant complaints with cosmetic and functional results [126]. Mental health and quality of life in women with 46,XY DSD who received early genitoplasty as young children have been shown to be no different than that of unaffected women [127]. Despite this limited support for early genitoplasty, it is not known if such beliefs generalize to patients and parents more widely. As already alluded to, some people with 46,XY DSD strongly oppose the decision that their parents and physicians made to proceed with surgery to “correct” their ambiguous genitalia as young children. While calls for changes in surgical standards of care for genital ambiguity including a moratorium on early genitoplasty have been requested by DSD advocates, [128] the current lack of information about the pros and cons of early genitoplasty such as systematic assessment of patient satisfaction with these procedures is delaying widespread changes in surgical standards of care for DSD. 

## 9. Summary and Conclusions

DSD results when discordance between a person's genetic sex, gonadal sex, external genitalia, and/or internal sex duct development exists at birth. A greater appreciation for the impact of hormones on behavior, unsatisfactory surgical outcomes related to DSD, and patient advocate positions have resulted in a reappraisal of medical and surgical treatments for DSD. One important component of this reappraisal is a call for better understanding of GI and GR development in individuals with 46,XY DSD, as well as an appreciation of the multiple factors beyond genes and hormones that contribute to psychosexual development. While it is not possible to study the influence of variables such as genes and hormone exposure independent of socialization and learning related to sex of rearing in most people, people with 46,XY DSD allow for such analyses. For example, a girl with CAIS possess a 46,XY chromosome complement (male pattern), no androgen exposure (female pattern) and female sex of rearing as described earlier. Based on the available literature, girls with this type of 46,XY DSD are expected to develop a female GI and GR. Therefore, it is possible to deduce from studies of girls and women with CAIS that possession of a Y chromosome has no significant impact on psychosexual development in humans; however, socialization and learning in the absence of early androgen exposure exert major influences on the establishment of gender. Studies of gender development in people with DSD not only contribute to better care and optimal outcomes in affected individuals, they also inform us about how genes, hormones and learning impact psychosexual development for all people.

In general, data from people with most forms of 46,XY DSD reveal that GI is predominantly influenced by learning and socialization associated with sex of rearing. This is particularly true for people with 46,XY DSD born with female external genitalia. However, genetic males with 5*α*-RD2 or 17*β*HSD-3 deficiency are the exception to this rule. Both of these androgen biosynthetic defects are associated with male GI development despite female rearing, even when the genitalia are female-typical. For the minority of people with 46,XY DSD who do develop a GI that is discordant with their sex of rearing, it is not known how early hormones and/or inconsistent or ambiguous socialization contributes to this outcome. Additionally, most data on GI development are collected from patients in Western societies. Further study of affected individuals from other cultures are needed. A better understanding of what causes a person to develop a GI that differs from their sex of rearing would result in improved processes for assigning a sex of rearing to newborns with DSD. In contrast, it is well documented that masculine patterns of GR are influenced by early androgen exposure that exceed what a typical female experiences. However, as girls and boys with 46,XY DSD mature into adolescence and adulthood, their GR pattern increasingly corresponds with their sex of rearing. Whether exposure to pubertal hormones and/or additive learning over time causes GR to increasingly correspond with sex of rearing is unknown.

Apart from studies of genetic and hormonal influences on gender in people with 46,XY DSD reared male or female, variables such as how parents react to their child's diagnosis including whether or not to proceed with early genitoplasty are important to consider. Typically, parents attempt to reduce their affected child's sexual ambiguity by reinforcing certain behaviors such as playmate preferences and occupational interests, as well as by opting for surgery to “correct” their child's genital ambiguity and remove the testes in children reared female. However, no data currently exist to support the notion that early surgery significantly impacts gender development in people with 46,XY DSD. Parents of children with these conditions view their children as vulnerable, and they sometimes report significant levels of stress associated with parenting their affected child. Recent questioning of the appropriateness of performing early genital surgeries on young girls and boys with 46,XY DSD may also contribute to parents' concerns. More data are needed to assess outcomes of early genitoplasty (both feminizing and masculinizing), including GI and GR development in people who do not receive these early surgeries, cosmetic and functional outcomes, ultimate patient satisfaction with surgery and quality of life in those who experience these procedures to resolve the ongoing debate about whether or not to change surgical standards of care for DSD. 

## 10. Future Directions of Medical Management and Research

While we have learned a great deal about many types of 46,XY DSD since the initial studies of patients conducted by John Money and his colleagues, starting in the 1950s and extending through the start of the 21st century, [31] much remains to be learned. Several key areas are ripe for improvements in the medical and surgical management of DSD. For example, approximately half of all patients with 46,XY DSD today have yet to receive a definitive diagnosis, [9] although patients who receive their medical care in multidisciplinary DSD clinics are more likely to obtain this information [104]. Advances in technology, coupled with the adoption of systematic protocols for examining patients, will hopefully ameliorate this problem [9]. Continued growth of multidisciplinary DSD clinics throughout the United States may be needed for this particular improvement to be realized. Additionally, patients and their families must receive complete, up-to-date information about the condition that affects them including treatment options that are available and the pros and cons associated with these [10]. An integral part to providing DSD care is to eliminate shame and secrecy that has so often been associated with these conditions in the past [9]. An excellent way to accomplish this is to provide patients with information about peer support groups so that they may communicate with others who share a common medical history. Examples of such groups include, but are not limited to, the organizations dsdfamilies, AISDSD, the MAGIC Foundation, and CARES. Additionally, eliminating shame and secrecy for patients and their families can be achieved by introducing parents of newborns with 46,XY DSD to parents of older affected children within a clinic. Doing so not only removes feelings of isolation for the new parents, but it also allows the parents who are more experienced with treatments such as genitoplasty to provide their opinion about whether or not proceeding with this surgical option was beneficial for their child. It is of interest that advances in technology are critical for improving diagnostic services, whereas open communication is all that is needed to optimize patient and parent education as well as to remove feelings of shame, secrecy, and isolation. 

Similar to medical care, an immense amount of information has been derived from research conducted over the last 6 decades concerning people with 46,XY DSD [9]. Such studies have revealed the natural history of GI and GR associated with specific conditions that are categorized as 46,XY DSD, as well as some of the variables that influence these 2 aspects of psychosexual development. In the past such studies have emphasized the role of early androgen exposure on gender. More recently, researchers have increasingly considered the impact of family members and peers on the behavioral development of children with 46,XY DSD as they mature into women and men. Because parents exert a primary influence on their children's psychological development, increased consideration of parents' reactions to DSD and related treatment options, as well as their parenting style with their affected children, is needed to more fully understand why some patients develop gender in an expected manner while others do not.

Whether or not early genitoplasty is beneficial or harmful to children with 46,XY DSD is an area of intense, continued debate that will only come to resolution when adequate genitoplasty outcome data are collected. To accomplish this, information about “good” cosmetic outcome and “satisfactory” sexual function needs to be operationalized and applied consistently to all people who receive such surgeries are being studied. As 46,XY DSD is rare, such studies require data collection across multiple hospitals that perform genitoplasty in children. Just as communication is the only requirement for increasing education about DSD for patients and families as well as decreasing feelings of isolation, shame, and secrecy, collaboration is all that is needed for collecting the data necessary to resolve the debate surrounding early genitoplasty.

Finally, an important future direction for advancing DSD research is to identify markers that predict a person's GI and GR independent of self-report and parent observation. Such a marker should reliably differ between male-typical and female-typical GI and GR and not be influenced by expectations of study subjects, their parents and doctors, or researchers. If such a marker was present and constant from birth onward, then it might be used as part of an algorithm for predicting psychosexual development. This would then eliminate mistakes in sex of rearing, pubertal induction, and genitoplasty that are experienced by a significant minority of people with 46,XY DSD, reared male or female.

## References

[1] Eugster EA (2004). Reality vs recommendations in the care of infants with intersex conditions. *Archives of Pediatrics and Adolescent Medicine*.

[2] Frader J, Alderson P, Asch A (2004). Health care professionals and intersex conditions. *Archives of Pediatrics and Adolescent Medicine*.

[3] Nelson CP, Gearhart JP (2004). Current views on evaluation, management, and gender assignment of the intersex infant. *Nature Clinical Practice Urology*.

[4] Diamond M, Beh HG (2008). Changes in the management of children with intersex conditions. *Nature Clinical Practice Endocrinology and Metabolism*.

[5] Feder EK (2009). Normalizing medicine: between “intersexuals” and individuals with ‘disorders of sex development’. *Health Care Analysis*.

[6] Wiesemann C (2010). Ethical guidelines for the clinical management of intersex. *Sexual Development*.

[7] Wiesemann C, Ude-Koeller S, Sinnecker GHG, Thyen U (2010). Ethical principles and recommendations for the medical management of differences of sex development (DSD)/intersex in children and adolescents. *European Journal of Pediatrics*.

[8] Melton L (2001). New perspectives on the management of intersex. *The Lancet*.

[9] Hughes IA, Houk C, Ahmed SF, Lee PA (2006). Consensus statement on management of intersex disorders. *Journal of Pediatric Urology*.

[10] Wisniewski AB, Chernausek SD, Kropp BP (2012). *Disorders of Sex Development: A Practical Guide for Parents and Physicians*.

[11] Diamond M (2011). Developmental, sexual and reproductive neuroendocrinology: historical, clinical and ethical considerations. *Frontiers in Neuroendocrinology*.

[12] Brain CE, Creighton SM, Mushtaq I (2010). Holistic management of DSD. *Best Practice and Research*.

[13] Lee PA, Houk CP (2010). The role of support groups, advocacy groups, and other interested in parties in improving the care of patients with congenital adrenal hyperplasia: pleas and warnings. *International Journal of Pediatric Endocrinology*.

[14] Arnhold IJP, Melo K, Costa EMF (2011). 46,XY disorders of sex development (46,XY DSD) due to androgen receptor defects: androgen insensitivity syndrome. *Advances in Experimental Medicine and Biology*.

[15] Gaspari L, Paris F, Philibert P (2011). ‘Idiopathic’ partial androgen insensitivity syndrome in 28 newborn and infant males: impact of prenatal exposure to environmental endocrine disruptor chemicals?. *European Journal of Endocrinology*.

[16] Mendonca BB, Costa EMF, Belgorosky A, Rivarola MA, Domenice S (2010). 46,XY DSD due to impaired androgen production. *Best Practice and Research*.

[17] Fluck C, Meyer-Böni M, Pandey A (2011). Why boys will be boys: two pathways for fetal testicular androgen biosynthesis are needed for male sexual differentiation. *American Journal of Human Genetics*.

[18] Morel Y, Plotton I, Mallet D (2011). Studies of a cohort of 46,XY with DSD including steroid biosynthesis deficiencies. *Advances in Experimental Medicine and Biology*.

[19] Köhler B, Lin L, Mazen I (2009). The spectrum of phenotypes associated with mutations in steroidogenic factor 1 (SF-1, NR5A1, Ad4BP) includes severe penoscrotal hypospadias in 46,XY males without adrenal insufficiency. *European Journal of Endocrinology*.

[20] Pearlman A, Loke J, Le Caignec C (2010). Mutations in MAP3K1 cause 46,XY disorders of sex development and implicate a common signal transduction pathway in human testis determination. *American Journal of Human Genetics*.

[21] Dai YL, Fu JF, Hong F, Xu S, Shen Z (2011). WT1 mutation as a cause of 46 XY DSD and Wilm’s tumour: a case report and literature review. *Acta Paediatrica, International Journal of Paediatrics*.

[22] Ferraz-de-Souza B, Lin L, Achermann JC (2011). Steroidogenic factor-1 (SF-1, NR5A1) and human disease. *Molecular and Cellular Endocrinology*.

[23] Knower KC, Kelly S, Ludbrook LM (2011). Failure of SOX9 regulation in 46XY disorders of sex development with *SRY*, SOX9 and SF1 mutations. *PLoS ONE*.

[24] Lourenço D, Brauner R, Rybczyńska M, Nihoul-Fékété C, McElreavey K, Bashamboo A (2011). Loss-of-function mutation in GATA4 causes anomalies of human testicular development. *Proceedings of the National Academy of Sciences of the United States of America*.

[25] Philibert P, Polak M, Colmenares A (2011). Predominant Sertoli cell deficiency in a 46,XY disorders of sex development patient with a new NR5A1/SF-1 mutation transmitted by his unaffected father. *Fertility and Sterility*.

[26] White S, Ohnesorg T, Notini A (2011). Copy number variation in patients with disorders of sex development due to 46,XY gonadal dysgenesis. *PLoS ONE*.

[27] Camats N, Pandey AV, Fernández-Cancio M (2012). Ten novel mutations in the NR5A1 gene cause disordered sex development in 46,XY and ovarian insufficiency in 46,XX individuals. *Journal of Clinical Endocrinology and Metabolism*.

[28] Bashamboo A, Ledig S, Wieacker P, Achermann JC, McElreavey K (2010). New technologies for the identification of novel genetic markers of disorders of sex development (DSD). *Sexual Development*.

[29] Arboleda V, Lee H, Sánchez F Targeted massively parallel sequencing provides comprehensive genetic diagnosis for patients with disorders of sex development.

[30] Schober JM (2001). Sexual behaviors, sexual orientation and gender identity in adult intersexuals: a pilot study. *Journal of Urology*.

[31] Money J, Ehrhardt AA (1972). *Man & Woman, Boy & Girl: The Differentiation and Dimorphism of Gender Identity from Conception to Maturity*.

[32] Didonato MD, Berenbaum SA (2011). The benefits and drawbacks of gender typing: how different dimensions are related to psychological adjustment. *Archives of Sexual Behavior*.

[33] DiDonato MD, Berenbaum SA Predictors and consequences of gender typicality: the mediating role of communality.

[34] Meyer-Bahlburg HFL (2011). Gender monitoring and gender reassignment of children and adolescents with a somatic disorder of sex development. *Child and Adolescent Psychiatric Clinics of North America*.

[35] Crissman HP, Warner L, Gardner M (2011). Children with disorders of sex development : a qualitative study of early parental experience. *International Journal of Pediatric Endocrinology*.

[36] De Vries ALC, Doreleijers TAH, Cohen-Kettenis PT (2007). Disorders of sex development and gender identity outcome in adolescence and adulthood: understanding gender identity development and its clinical implications. *Pediatric Endocrinology Reviews*.

[37] Jürgensen M, Hiort O, Holterhus PM, Thyen U (2007). Gender role behavior in children with XY karyotype and disorders of sex development. *Hormones and Behavior*.

[38] Bradley SJ, Oliver GD, Chernick AB, Zucker KJ (1998). Experiment of nurture: ablatio penis at 2 months, sex reassignment at 7 months, and a psychosexual follow-up in young adulthood. *Pediatrics*.

[39] Kuhnle U, Krahl W (2002). The impact of culture on sex assignment and gender development in intersex patients. *Perspectives in Biology and Medicine*.

[40] Warne GL (2008). Long-term outcome of disorders of sex development. *Sexual Development*.

[41] Ismail SI, Mazen IA (2010). A study of gender outcome of Egyptian patients with 46,XY disorder of sex development. *Sexual Development*.

[42] Al Jurayyan NAM (2011). Disorders of sex development: diagnostic approaches and management options-an Islamic perspective. *Malaysian Journal of Medical Sciences*.

[43] Pappas KB, Wisniewski AB, Migeon CJ (2008). Gender role across development in adults with 46,XY disorders of sex development including perineoscrotal hypospadias and small phallus raised male or female. *Journal of Pediatric Endocrinology and Metabolism*.

[44] Wisniewski AB, Mazur T (2009). 46,XY DSD with female or ambiguous external genitalia at birth due to androgen insensitivity syndrome, 5alpha-reductase-2 deficiency, or 17beta-hydroxysteoid dehydrogenase deficiency: a review of quality of life outcomes. *International Journal of Pediatric Endocrinology*.

[45] Praveen EP, Desai AK, Khurana ML (2008). Gender identity of children and young adults with 5*α*-reductase deficiency. *Journal of Pediatric Endocrinology and Metabolism*.

[46] Uslu R, Oztop D, Yilmaz S (2007). Factors contributing to sex assignment and reassignment decisions in Turkish children with 46,XY disorders of sex development. *Journal of Pediatric Endocrinology and Metabolism*.

[47] Probst R, Lonsbury-Martin BL, Martin GK (1991). A review of otoacoustic emissions. *Journal of the Acoustical Society of America*.

[48] Bilger RC, Matthies ML, Hammel DR, Demorest ME (1990). Genetic implications of gender differences in the prevalence of spontaneous otoacoustic emissions. *Journal of Speech and Hearing Research*.

[49] McFadden D, Loehlin JC, Pasanen EG (1996). Additional findings on heritability and prenatal masculinization of cochlear mechanisms: click-evoked otoacoustic emissions. *Hearing Research*.

[50] McFadden D (1998). Sex differences in the auditory system. *Developmental Neuropsychology*.

[51] McFadden D (2002). Masculinization effects in the auditory system. *Archives of Sexual Behavior*.

[52] McFadden D, Pasanen EG, Weldele ML, Glickman SE, Place NJ (2006). Masculinized otoacoustic emissions in female spotted hyenas (*Crocuta crocuta*). *Hormones and Behavior*.

[53] McFadden D, Pasanen EG (1999). Spontaneous otoacoustic emissions in heterosexuals, homosexuals, and bisexuals. *Journal of the Acoustical Society of America*.

[54] Hughes IA, Davies JD, Bunch TI, Pasterski V, Mastroyannopoulou K, MacDougall J (2012). Androgen insensitivity syndrome. *The Lancet*.

[55] Gottlieb B, Beitel LK, Nadarajah A, Paliouras M, Trifiro M (2004). The androgen receptor gene mutations database: 2012 update. *Human Mutation*.

[56] Masica DN, Money J, Ehrhardt AA (1971). Fetal feminization and female gender identity in the testicular feminizing syndrome of androgen insensitivity. *Archives of Sexual Behavior*.

[57] Wisniewski AB, Migeon CJ, Meyer-Bahlburg HFL (2000). Complete androgen insensitivity syndrome: long-term medical, surgical, and psychosexual outcome. *Journal of Clinical Endocrinology and Metabolism*.

[58] Hines M, Ahmed SF, Hughes IA (2003). Psychological outcomes and gender-related development in complete androgen insensitivity syndrome. *Archives of Sexual Behavior*.

[59] Melo KFS, Mendonca BB, Billerbeck AEC (2003). Clinical, hormonal, behavioral, and genetic characteristics of androgen insensitivity syndrome in a Brazilian cohort: five novel mutations in the androgen receptor gene. *Journal of Clinical Endocrinology and Metabolism*.

[60] Hooper HT, Figueiredo BC, Pavan-Senn CC (2004). Concordance of phenotypic expression and gender identity in a large kindred with a mutation in the androgen receptor. *Clinical Genetics*.

[61] Brinkmann L, Schuetzmann K, Richter-Appelt H (2007). Gender assignment and medical history of individuals with different forms of intersexuality: evaluation of medical records and the patients’ perspective. *Journal of Sexual Medicine*.

[62] Richter-Appelt H, Discher C, Gedrose B (2005). Gender identity and recalled gender related childhood play-behaviour in adult individuals with different forms of intersexuality. *Anthropologischer Anzeiger; Bericht über die biologisch-anthropologische Literatur*.

[63] T’Sjoen G, De Cuypere G, Monstrey S (2011). Male gender identity in complete androgen insensitivity syndrome. *Archives of Sexual Behavior*.

[64] Mazur T (2005). Gender dysphoria and gender change in androgen insensitivity or micropenis. *Archives of Sexual Behavior*.

[65] Migeon CJ, Wisniewski AB, Gearhart JP (2002). Ambiguous genitalia with perineoscrotal hypospadias in 46,XY individuals: long-term medical, surgical, and psychosexual outcome. *Pediatrics*.

[66] Jurgensen M, Kleinemeier E, Lux A Psychosexual development in adolescents and adults with disorders of sex development—results from the German Clinical Evaluation Study.

[67] Cohen-Kettenis P (2005). Psychological long-term outcome in intersex conditions. *Hormone Research*.

[68] Mendonca BB, Inacio M, Costa EMF (1996). Male pseudohermaphroditism due to steroid 5*α*-reductase 2 deficiency: diagnosis, psychological evaluation, and management. *Medicine*.

[69] Al-Attia HM (1996). Gender identity and role in a pedigree of Arabs with intersex due to 5 alpha reductase-2 deficiency. *Psychoneuroendocrinology*.

[70] Sobel V, Imperato-McGinley J (2004). Gender identity in XY intersexuality. *Child and Adolescent Psychiatric Clinics of North America*.

[71] Cheon CK (2011). Practical approach to steroid 5alpha-reductase type 2 deficiency. *European Journal of Pediatrics*.

[72] Uehara S, Hashiyada M, Sato K, Nata M, Funato T, Okamura K (2002). Complete XY gonadal dysgenesis and aspects of the *SRY* genotype and gonadal tumor formation. *Journal of Human Genetics*.

[73] Bagci G, Bisgin A, Karauzum SB, Trak B, Luleci G (2011). Complete gonadal dysgenesis 46,XY (Swyer syndrome) in two sisters and their mother’s maternal aunt with a female phenotype. *Fertility and Sterility*.

[74] Doherty LF, Rackow BW (2011). Abnormal streak gonads in 46,XY complete gonadal dysgenesis. *Fertility and Sterility*.

[75] Rocha VBC, Guerra-Júnior G, Marques-De-Faria AP, De Mello MP, Maciel-Guerra AT (2011). Complete gonadal dysgenesis in clinical practice: the 46,XY karyotype accounts for more than one third of cases. *Fertility and Sterility*.

[76] Bianco S, Agrifoglio V, Mannino F, Cefalu E, Cittadini E (1992). Successful pregnancy in a pure gonadal dysgenesis with karyotype 46,XY patient (Swyer’s syndrome) following oocyte donation and hormonal treatment. *Acta Europaea Fertilitatis*.

[77] Siddique H, Daggett P, Artley K (2008). Successful term vaginal delivery in a 46,XY woman. *International Journal of Gynecology and Obstetrics*.

[78] McCarty BM, Migeon CJ, Meyer-Bahlburg HFL, Zacur H, Wisniewski AB (2006). Medical and psychosexual outcome in women affected by complete gonadal dysgenesis. *Journal of Pediatric Endocrinology and Metabolism*.

[79] McElreavey K, Vilain E, Barbaux S (1996). Loss of sequences 3′ to the testis-determining gene, *SRY*, including the Y pseudoautosomal boundary associated with partial testicular determination. *Proceedings of the National Academy of Sciences of the United States of America*.

[80] Barbaro M, Cicognani A, Balsamo A (2008). Gene dosage imbalances in patients with 46,XY gonadal DSD detected by an in-house-designed synthetic probe set for multiplex ligation-dependent probe amplification analysis. *Clinical Genetics*.

[81] de Mello MP, Coeli FB, Assumpção JG (2010). Novel DMRT1 3’UTR+11insT mutation associated to XY partial gonadal dysgenesis. *Arquivos Brasileiros de Endocrinologia e Metabologia*.

[82] Rey RA, Belville C, Nihoul-Fékété C (1999). Evaluation of gonadal function in 107 intersex patients by means of serum antimullerian hormone measurement. *Journal of Clinical Endocrinology and Metabolism*.

[83] Szarras-Czapnik M, Lew-Starowicz Z, Zucker KJ (2007). A psychosexual follow-up study of patients with mixed or partial gonadal dysgenesis. *Journal of Pediatric and Adolescent Gynecology*.

[84] Wilson JD (2001). Androgens, androgen receptors, and male gender role behavior. *Hormones and Behavior*.

[85] Beltz AM, Swanson JL, Berenbaum SA (2011). Gendered occupational interests: prenatal androgen effects on psychological orientation to Things versus People. *Hormones and Behavior*.

[86] Hines M (2011). Prenatal endocrine influences on sexual orientation and on sexually differentiated childhood behavior. *Frontiers in Neuroendocrinology*.

[87] Berenbaum SA, Duck SC, Bryk K (2000). Behavioral effects of prenatal versus postnatal androgen excess in children with 21-hydroxylase-deficient congenital adrenal hyperplasia. *Journal of Clinical Endocrinology and Metabolism*.

[88] Cohen-Bendahan CCC, Van De Beek C, Berenbaum SA (2005). Prenatal sex hormone effects on child and adult sex-typed behavior: methods and findings. *Neuroscience and Biobehavioral Reviews*.

[89] Frisén L, Nordenström A, Falhammar H (2009). Gender role behavior, sexuality, and psychosocial adaptation in women with congenital adrenal hyperplasia due to CYP21A2 deficiency. *Journal of Clinical Endocrinology and Metabolism*.

[90] Meyer-Bahlburg HFL, Dolezal C, Baker SW, Ehrhardt AA, New MI (2006). Gender development in women with congenital adrenal hyperplasia as a function of disorder severity. *Archives of Sexual Behavior*.

[91] Maccoby EE, Jacklin CN, Reece HW (1987). Gender segregation in children. *Advances in Child Development and Behavior*.

[92] Golombok S, Hines M, Smith PK, Hart SH (2002). Sex differences in social behavior. *Handbook of Childhood Sexual Development*.

[93] Drotar D (1997). Relating parent and family functioning to the psychological adjustment of children with chronic health conditions: what have we learned? What do we need to know?. *Journal of Pediatric Psychology*.

[94] Sanders C, Carter B, Goodacre L (2008). Parents’ narratives about their experiences of their child’s reconstructive genital surgeries for ambiguous genitalia. *Journal of Clinical Nursing*.

[95] Slijper FME, Frets PG, Boehmer ALM, Drop SLS, Niermeijer MF (2000). Androgen insensitivity syndrome (AIS): emotional reactions of parents and adult patients to the clinical diagnosis of AIS and its confirmation by androgen receptor gene mutation analysis. *Hormone Research*.

[96] Wysocki T, Gavin L (2006). Paternal involvement in the management of pediatric chronic diseases: associations with adherence, quality of life, and health status. *Journal of Pediatric Psychology*.

[97] Sanders C, Carter B, Goodacre L (2011). Searching for harmony: parents’ narratives about their child’s genital ambiguity and reconstructive genital surgeries in childhood. *Journal of Advanced Nursing*.

[98] Wong WI, Pasterski V, Hindmarsh PC, Geffner ME, Hines M Are there parental socialization effects on the sex-typed behavior of individuals with congenital adrenal hyperplasia?.

[99] Sanders C, Carter B, Goodacre L Parents need to protect: influences, risks and tensions for parents of prepubertal children born with ambiguous genitalia.

[100] Duguid A, Morrison S, Robertson A, Chalmers J, Youngson G, Ahmed SF (2007). The psychological impact of genital anomalies on the parents of affected children. *Acta Paediatrica, International Journal of Paediatrics*.

[101] Kirk KD, Fedele DA, Wolfe-Christensen C (2010). Parenting characteristics of female caregivers of children affected by chronic endocrine conditions: a comparison between disorders of sex development and type 1 diabetes mellitus. *Journal of Pediatric Nursing*.

[102] Fedele DA, Kirk K, Wolfe-Christensen C (2010). Primary caregivers of children affected by disorders of sex development: mental health and caregiver characteristics in the context of genital ambiguity and genitoplasty. *International Journal of Pediatric Endocrinology*.

[103] Hullmann SE, Fedele DA, Wolfe-Christensen C, Mullins LL, Wisniewski AB (2011). Differences in adjustment by child developmental stage among caregivers of children with disorders of sex development. *International Journal of Pediatric Endocrinology*.

[104] Palmer BW, Wisniewski AB, Schaeffer TL (2012). A model of delivering multi-disciplinary care to people with 46 XY DSD. *Journal of Pediatric Urology*.

[105] Cools M, Looijenga LHJ, Wolffenbuttel KP, Drop SLS (2009). Disorders of sex development: update on the genetic background, terminology and risk for the development of germ cell tumors. *World Journal of Pediatrics*.

[106] Cheikhelard A, Morel Y, Thibaud E (2008). Long-term followup and comparison between genotype and phenotype in 29 cases of complete androgen insensitivity syndrome. *Journal of Urology*.

[107] Allen L (2009). Opinion one: a case for delayed gonadectomy. *Journal of Pediatric and Adolescent Gynecology*.

[108] Hurt WG, Bodurtha JN, McCall JB, Moinuddin Ali M (1989). Seminoma in pubertal patient with androgen insensitivity syndrome. *American Journal of Obstetrics and Gynecology*.

[109] Kang HJ, Imperato-Mcginley J, Zhu YS (2011). The first successful paternity through in vitro fertilization- intracytoplasmic sperm injection with a man homozygous for the 5*α*-reductase-2 gene mutation. *Fertility and Sterility*.

[110] Clayton PE, Oberfield SE, Martin Ritzén E (2002). Consensus: consensus statement on 21-hydroxylase deficiency from The Lawson Wilkins Pediatric Endocrine Society and The European Society for Pediatric Endocrinology. *Journal of Clinical Endocrinology and Metabolism*.

[111] Wisniewski AB, Migeon CJ, Gearhart JP (2001). Congenital micropenis: long-term medical, surgical and psychosexual follow-up of individuals raised male or female. *Hormone Research*.

[112] Meyer-Bahlburg HFL, Migeon CJ, Berkovitz GD, Gearhart JP, Dolezal C, Wisniewski AB (2004). Attitudes of adult 46,XY intersex persons to clinical management policies. *Journal of Urology*.

[113] Palmer BW, Reiner W, Kropp BP (2012). Proximal hypospadias repair outcomes in patients with a specific disorder of sexual development diagnosis. *Advances in Urology*.

[114] Wilson JM, Arnhym A, Champeau A, Ebbers M, Coakley F, Baskin L (2011). Complete androgen insensitivity syndrome: an anatomic evaluation and sexual function questionnaire pilot study. *Journal of Pediatric Urology*.

[115] Fagerholm R, Santtila P, Miettinen PJ, Mattila A, Rintala R, Taskinen S (2011). Sexual function and attitudes toward surgery after feminizing genitoplasty. *Journal of Urology*.

[116] Köhler B, Kleinemeier E, Lux A (2012). Satisfaction with genital surgery and sexual life of adults with XY disorders of sex development: results from the german clinical evaluation study. *Journal of Clinical Endocrinology and Metabolism*.

[117] Nihoul-Fékété C, Thibaud E, Lortat-Jacob S, Josso N (2006). Long-Term Surgical Results and Patient Satisfaction With Male Pseudohermaphroditism or True Hermaphroditism: a Cohort of 63 Patients. *Journal of Urology*.

[118] Pasterski V, Prentice P, Hughes IA (2010). Consequences of the Chicago consensus on disorders of sex development (DSD): current practices in Europe. *Archives of Disease in Childhood*.

[119] Douglas G, Axelrad ME, Brandt ML (2012). Guidelines for evaluating and managing children born with disorders of sexual Development. *Pediatric Annals*.

[120] Schaeffer TL, Tryggestad JB, Mallappa A, Hanna AE, Krishnan S, Chernausek SD (2010). An evidence-based model of multidisciplinary care for patients and families affected by classical congenital adrenal hyperplasia due to 21-hydroxylase deficiency. *International Journal of Pediatric Endocrinology*.

[121] Moran ME, Karkazis K (2012). Developing a multidisciplinary team for disorders of sex development: planning, implementation, and operation tools for care providers. *Advances in Urology*.

[122] Streuli JC, Köhler B, Werner-Rosen K, Mitchell C (2012). DSD and professionalism from a multilateral view: supplementing the consensus statement on the basis of a qualitative survey. *Advances in Urology*.

[123] Kogan BA, Gardner M, Alpern AN (2012). Challenges of disorders of sex development: diverse perceptions across stakeholders. *Hormone Research in Paediatrics*.

[124] Hurwitz RS (2011). Feminizing surgery for disorders of sex development: evolution, complications, and outcomes. *Current Urology Reports*.

[125] Nihoul-Fékété C (2005). Does surgical genitoplasty affect gender identity in the intersex infant?. *Hormone Research*.

[126] Sircili MHP, De Queiroz E Silva FA, Costa EMF (2010). Long-term surgical outcome of masculinizing genitoplasty in large cohort of patients with disorders of sex development. *Journal of Urology*.

[127] Fagerholm R, Mattila AK, Roine RP, Sintonen H, Taskinen S (2012). Mental health and quality of life after feminizing genitoplasty. *Journal of Pediatric Surgery*.

[128] Diamond M (2004). Sex, gender, and identity over the years: a changing perspective. *Child and Adolescent Psychiatric Clinics of North America*.

